# Sex-Dependent Effects on Liver Inflammation and Gut Microbial Dysbiosis After Continuous Developmental Exposure to Trichloroethylene in Autoimmune-Prone Mice

**DOI:** 10.3389/fphar.2020.569008

**Published:** 2020-10-29

**Authors:** Sarah J. Blossom, Kuppan Gokulan, Matthew Arnold, Sangeeta Khare

**Affiliations:** ^1^ Department of Pediatrics, University of Arkansas for Medical Sciences and Arkansas Children’s Research Institute, Little Rock, AR, United States; ^2^ Division of Microbiology, National Center for Toxicological Research, US Food and Drug Administration, Jefferson, AR, United States

**Keywords:** trichloroethylene, autoimmune, liver, microbiome, gut, inflammation, CD4^+^ T cells

## Abstract

Trichloroethylene (TCE) is a common environmental toxicant linked with hypersensitivity and autoimmune responses in humans and animal models. While autoimmune diseases are more common in females, mechanisms behind this disparity are not clear. Recent evidence suggests that autoimmunity may be increasing in males, and occupational studies have shown that TCE-mediated hypersensitivity responses occur just as often in males. Previous experimental studies in autoimmune-prone MRL^+/+^ mice have focused on responses in females. However, it is important to include both males and females in order to better understand sex-disparity in autoimmune disease. In addition, because of an alarming increase in autoimmunity in adolescents, developmental and/or early life exposures to immune-enhancing environmental pollutants should also be considered. Using MRL^+/+^ mice, we hypothesized that TCE would alter markers related to autoimmunity to a greater degree in female mice relative to male mice, and that TCE would enhance these effects. Mice were continuously exposed to either TCE or vehicle beginning at gestation, continuing during lactation, and directly in the drinking water. Both male and female offspring were evaluated at 7 weeks of age. Sex-specific effects were evident. Female mice were more likely than males to show enhanced CD4^+^ T cell cytokine responses (e.g., IL-4 and IFN-γ). Although none of the animals developed pathological or serological signs of autoimmune hepatitis-like disease, TCE-exposed female mice were more likely than males in either group to express higher levels of biomarkers in the liver related to regeneration/repair and proliferation. Levels of bacterial populations in the intestinal ileum were also altered by TCE exposure and were more prominent in females as compared to males. Thus, our expectations were correct in that young adult female mice developmentally exposed to TCE were more likely to exhibit alterations in immunological and gut/liver endpoints compared to male mice.

## Introduction

Trichloroethylene **(**TCE) is a halocarbon best known for its use as an industrial solvent. Because of improper disposal, TCE has contaminated many water systems. TCE is among the most frequently detected US EPA-regulated drinking water contaminants found in 4.5% of groundwater and 15% of surface water sources (ATSDR, 2014). TCE’s effects on human health have been studied extensively. Recently TCE was classified as a human carcinogen (Guha et al., 2012). Among the myriad non-cancer outcomes associated with TCE exposure in humans is immunotoxicity. Chronic TCE exposure has been linked to autoimmune diseases including lupus, scleroderma, and autoimmune hepatitis (AIH) (Cooper et al., 2009; Parks and De Roos, 2014; Zhao et al., 2016).

Occupational studies have provided insight into health effects related to TCE exposure, because exposure levels are often known and thus easier to document. TCE exposure was associated with altered numbers of naïve and effector/memory CD4s (Hosgood et al., 2012) commensurate with decreased serum levels of IL-10 and IL-4, and increased IL-2 and IFN-γ reflective of a pro-inflammatory Th1 phenotype (Iavicoli et al., 2005; Bassig et al., 2013). In a recent investigation, workplace exposure to TCE well below the OSHA-mandated 8-h time weighted average altered immunological markers in the blood of exposed workers (Lee et al., 2019). Human occupational exposure has also been linked to DNA methylation changes in genes related to autoimmune disease (Phillips et al., 2019). Clinically, hypersensitivity responses have been documented in exposed workers (Kamijima et al., 2007). In extreme cases, some individuals developed a syndrome with severe skin lesions ranging from erythema to exfoliative dermatitis along with toxic epidermal necrolysis accompanying lymphadenopathy and fever. These diverse symptoms were often accompanied by immune-mediated liver injury often termed non-viral hepatitis (Dai et al., 2015). Although the liver manifestations appeared to be T cell-driven, it is not clear whether the immune response was directed toward self as in AIH.

Immune and liver effects similar to those observed in humans exposed to TCE have been investigated in animal models. TCE-induced immune-mediated liver injury was detected in Balb/c mice following acute dermal sensitization (Zhang et al., 2018; Wang et al., 2019b). Long-term exposure to TCE in drinking water of adult autoimmune-prone MRL^+/+^ mice promoted CD4-cell-mediated liver pathology similar to idiopathic AIH (Griffin et al., 2000; Cai et al., 2007; Gilbert et al., 2009). This TCE-induced pathology was associated with antibodies specific for liver microsomal proteins much like those observed in patients with AIH (Toh, 2017). TCE increased levels of mRNAs in the liver that encode for proteins involved in IL-6 signaling including IL-6 receptor, glycoprotein (gp) 130, a transmembrane signaling protein, and early growth receptor-1 (Egr-1) known to be important in liver regeneration and repair (Gilbert et al., 2014) and other non-TCE liver injury models (Wuestefeld et al., 2003). In addition to adult-only exposure similar serological and liver alterations were reported after developmental exposures (Gilbert et al., 2017; Blossom et al., 2018).

Aside from the liver, the gut is also involved in xenobiotic adsorption and metabolism. Impairment of the gut-liver axis is increasingly considered to play a role in the progression of inflammatory diseases. The liver receives most of its blood supply from the gut via the hepatic portal vein circulation. Consequently, the liver is continuously exposed to gut-derived factors including bacterial products (e.g., short-chain fatty acids), and both adaptive and innate immune components and their cytokines/chemokines (Seki and Schnabl, 2012). Thus, any modification in the balance between microbiota and gut immunological components may play a role in autoimmune diseases including AIH (Yuksel et al., 2015). In particular, an imbalance in proinflammatory/anti-inflammatory cells (López et al., 2016) and the development of ANAs was affected by the composition of gut microbiota, which was evident in early life (Van Praet et al., 2015).

Developmental TCE exposure was associated with decreased microbial abundance and a shift in microbial phyla [(*Bacteroidetes* to *Firmicutes*; (Blossom et al., 2018). Exposure to TCE during the developmental stage similarly had irreversible effects (i.e., after TCE removal during adulthood) on the gut microbial community and gut-associated immune response in adult MRL mice (Khare et al., 2019). In our model of TCE-induced AIH in lupus-prone female mice, CD4 cells infiltrated the liver leading to a cascade of inflammatory events that are counter balanced by increased IL-6 signaling. Similar findings have been reported in idiopathic AIH in humans (Nguyen Canh et al., 2017). Collectively, these findings support the concept that many factors interact to promote increased permeability of the GI mucosal barrier and translocation of gut-derived microbial and host mediators into the liver promoting AIH-like pathology.

Despite the numerous evaluations of this chemical, the effect of TCE on immune/gut/liver outcomes in males have not been characterized in experimental models. While autoimmune diseases disproportionally affect females (Jacobson et al., 1997; Fairweather and Rose, 2004), it has been suggested that males are just as likely as females to develop toxicant-induced autoimmunity (Pollard, 2012). A recent study using US National Health and Nutrition Examination Survey (NHANES) data found that autoimmunity appears to be increasing in adolescents, including males (Dinse et al., 2020). These studies strongly suggested that males needed to be included in experimental evaluations of chemically-induced autoimmunity. Because of this alarming increase in autoimmune markers in adolescents, developmental and/or early life exposures should also be considered. In the current study, we employed a basic experimental design of continuous developmental exposure with a single dose of TCE beginning at gestation as described (Meadows et al., 2017). Both male and female offspring were evaluated at the relatively young age of 7 weeks. We expected to find that TCE altered immune, liver, and gut markers to a greater degree in female relative to male mice, and that TCE would enhance these effects in both sexes.

## Materials and Methods

### Animals, Treatment, and Experimental Design

This study was approved by the Institutional Animal Care and Use Committee at the University of Arkansas for Medical Sciences. Adult male and female MRL^+/+^ mouse breeding pairs (6–8 weeks-of-age) were purchased from Jackson Laboratories (Bar Harbor, ME). Continuous developmental exposure to TCE has been described earlier (Meadows et al., 2017). Gestational day 0 (GD0) female mice were divided (following stratified randomization) into two treatment groups. The treated mice received 0.5 mg/ml TCE (purity 99^+^% purchased from Sigma, St. Louis, MO) mixed in ultrapure unchlorinated water. The dose of TCE that was selected for the current study has been shown to induce AIH-like tissue pathology as well as early signs of liver inflammation in older adult mice after both adult-only and developmental exposure (Gilbert et al., 2009). TCE-containing water was mixed with 1% Alkamuls EL-620, an emulsifier consisting of ethoxylated castor oil (Rhone-Poulenc, Cranbury, NJ) used to solubilize the TCE. Controls were administered ultrapure unchlorinated water with 1% Alkamuls EL-20 alone (vehicle control). Food was provided ad libitum.

**FIGURE 1:**
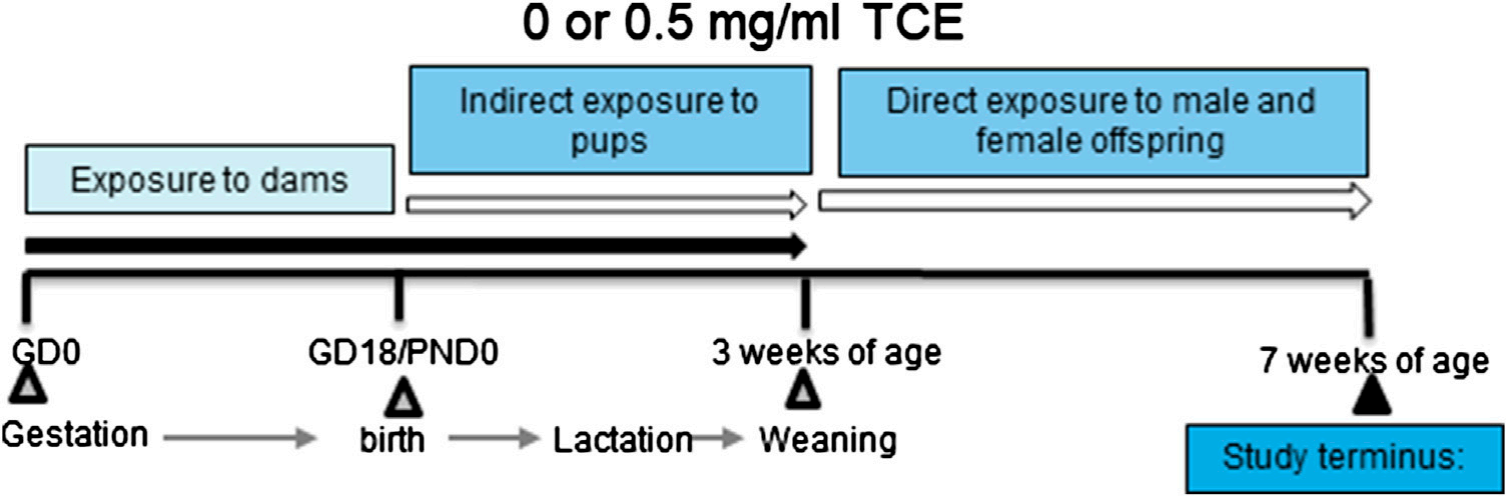
Experimental design. Female MRL^+/+^ mice were randomly assigned to one of two treatment groups (10 mice/treatment group). Each group consisted of either vehicle or trichloroethylene (TCE). All exposures began at gestation and continued during gestation and lactation. Female and male offspring were weaned at 3 weeks and exposed to the same treatment directly for an additional 4 weeks after which TCE was removed from the drinking water. Mice were euthanized at 7 weeks of age and tissues/cells assessed for parameters as described in detail in the *Materials and Methods* section.

The experimental design is depicted in Figure 1. Dams in each treatment group produced ten litters and all offspring remained with each dam until weaning. Maternal exposure to TCE or vehicle control containing drinking water continued throughout birth and lactation. Once pups were weaned at postnatal day 21 (PND21) their exposure to vehicle control or TCE directly in their drinking water continued for the duration of the experiment until euthanasia at ∼PND49. Upon study termination, tissues and cells from 4 to 10 randomly selected male and female offspring per litter were immediately removed and either processed immediately or snap frozen and stored at −80°C until assay.

### Liver RNA Isolation for qRT-PCR

Frozen liver was added to a tube filled with 0.8–1.0 ml Trizol Reagent (Life Technologies). Tissue was homogenized and subjected to chloroform and 2-isopropanol treatment to extract RNA according to established protocols. After centrifugation, pellets were washed with 70% ethanol and left to dry for 1 min. After dissolving in nuclease-free water, RNA was stored in RNase-free microcentrifuge tubes at −80°C until qRT-PCR.

### CD4 T Cell Isolation and Activation for qRT-PCR

CD4 T cells were isolated from spleen cell suspensions using a Dynabeads FlowComp mouse CD4 kit (Invitrogen). CD4 cells were seeded into 24-well plates and stimulated with immobilized anti-CD3 mAb and soluble anti-CD28 mAb for 18 h. CD4 cells were harvested, centrifuged, and reconstituted in a solution of RLT buffer (RNeasy, Qiagen) prior to RNA extraction, and subjected to the same protocol for RNA purification as described for liver. The RNA was frozen at −80°C until qRT-PCR.

### qRT-PCR

mRNA levels were analyzed by qRT-PCR using RNA isolated from CD4 cells and liver using the High Capacity cDNA Reverse transcriptase kit (Applied Biosystems) mRNA was quantified using Bio-Rad iTaq SYBR Green Supermix Primers procured from Integrated DNA Technologies. Samples were run in duplicate and averaged to obtain a mean fold change expression level and compared with the housekeeping control gene (EEF2) using 2^−ΔΔCt^ method.

### Western Blot Analysis

Sucrose buffer containing protease inhibitor cocktail (Thermo Fisher Scientific, Waltham, MA) was used to lyse liver tissue. Protein concentration was measured in homogenates using a BCA Protein Assay Kit (Thermo Fisher). Forty micrograms of homogenate per sample was separated by SDS polyacrylamide gel electrophoresis using 4–15% Mini-Protean Protein Gels (Bio-Rad, Hercules, CA). Gels were transferred to Hybond-ECL nitrocellulose membranes (Amersham Biosciences). After soaking in blocking buffer (5% milk in 1× PBS) for 3 h at 25°C, and washed in Tris buffered saline, membranes were incubated with primary antibody specific for CYP ^450^P 2E1 (Rabbit polyclonal; ab28146, purchased from Abcam) at a dilution of 1:1,000. Polyclonal antibody to detect housekeeping protein, GAPDH, was purchased from Cell Signaling Technology (Danvers, MA) and applied at a 1:1,000 dilution. Secondary HRP-conjugated polyclonal goat anti-rabbit IgG (1:1,000) was added. Immuonoreactions were detected with SuperSignal West Pico Chemiluminescent Substrate (Thermo Fisher) and developed with Hyperfilm ECL (Amersham Biosciences). Densitometric analysis (expressed in OD) was used to estimate CYP2E1 levels and normalized with expression levels of GAPDH.

### Detection of Gut Cytokines

Ileal tissues were harvested and flash frozen in liquid nitrogen and stored at −80°C prior to assay. Ileal tissues were removed from storage, weighed, lyzed, and extracted using a gentleMACS dissociator. After centrifugation (4°C for 10 min, 750 rpm), supernatant was transferred to Eppendorf tubes and centrifuged again (15 min, 10,000 × *g*). Protein concentration was measured in the spectrophotometer using Bio-Rad Protein Assay. All samples were diluted to a final concentration of 900 μg/ml and stored at −80°C until use for cytokine analysis as described (Gokulan et al., 2016). Twenty cytokines relevant to mucosal immunity were measured (IL-1α, IL-1β, IL-2, IL-3, IL-4, IL-5, IL-6, IL-10, IL-12p40, IL-12p70, IL-13, IL-17, Eotaxin, GM-CSF, IFN-γ, KC, MCP-1, MIP-1α, RANTES, and TNF-α) in duplicate using Bio-Plex Mouse Cytokine multiplex kits (Bio-Rad) per the manufacturer’s instructions.

### Identification of Bacterial Groups from Ileal Tissue

Ileal tissue lysate was prepared by bead-beating in a Fast Prep Machine. Proteins and RNA were removed by incubating with proteinase K, followed by a second incubation at 37°C for 15 min with RNase-A. An equal volume of phenol-chloroform-isopropanol was added to the tube containing lysate and mixed. After centrifuging at 12,000 × *g* for 30 min, the aqueous layer was removed and transferred to a new tube. DNA was precipitated using standard techniques. The cell pellet was washed with 70% ethanol and air dried. DNA was suspended and stored in nuclease-free water. Real-time PCR was used to amplify DNA fragments of bacteria using an ABI 7500 machine. Primer Express software (Applied Biosystems, Foster City, CA) was used to design primers for the identification of predominant phyla and representative genera and species of bacteria present in intestinal mucosa as previously reported (Williams et al., 2015). A list of primers used to quantify bacteria are included in **Supplementary Table S1**.

### Statistical Analysis

Assays were conducted using samples from four to eight individual mice in each treatment group. Summary statistics such as mean and SD are presented for each treatment group. Data were initially evaluated with a two-way ANOVA with interactions to determine the overall effects of TCE and sex, and the interaction between the two main factors. All analyses were followed by Tukey’s *post-hoc* tests to protect the overall significance level of 0.05. In order to compare responses among all groups of animals, six pre-specified pairwise comparisons included 1) male control vs. female control; 2) male control vs. male TCE; 3) male control vs. female TCE; 4) female control vs. male TCE; 5) female control vs. female TCE, and 6) male TCE vs. female TCE. Statistical significance resulting from these comparisons are reported in the graphs and the tables. All analyses were completed in GraphPad Prism 7.0 (LaJolla, CA).

## Results

### Increased Body Weight in Male Relative to Female Mice

**FIGURE 2:**
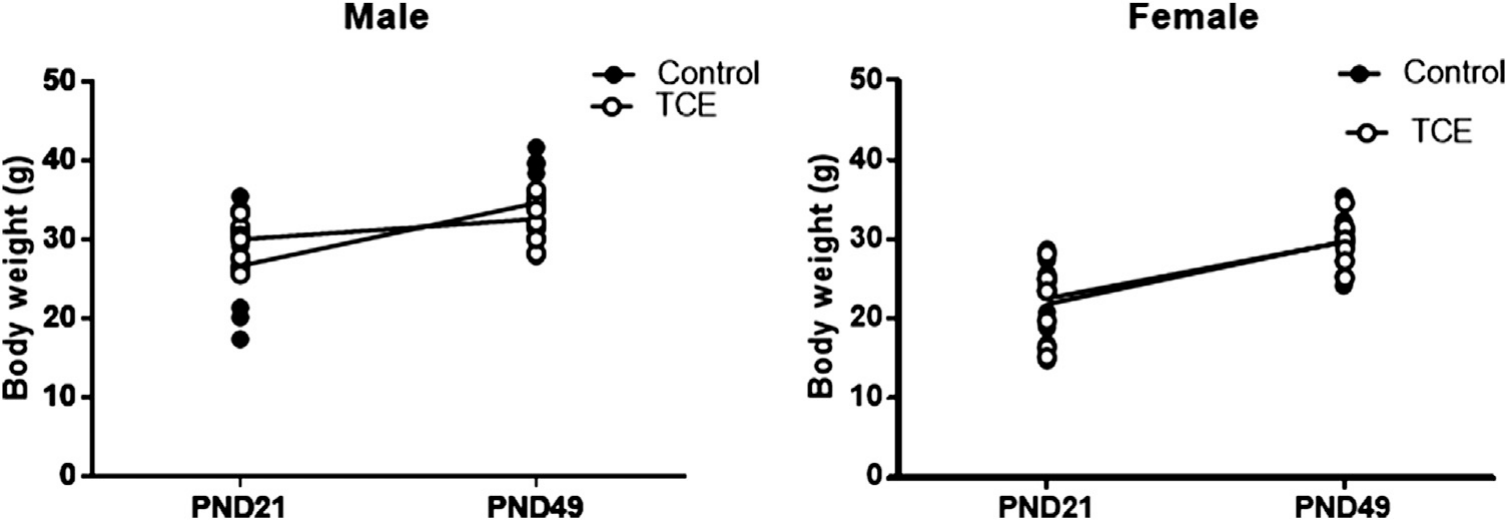
Mice were weighed once weekly starting at weaning. Body weights of the offspring are represented in grams and are shown at both weaning (3 weeks of age) and at study terminus (7 weeks of age). Each data point in the graph represents an individual mouse (1 male or female mouse per litter; *n* = 8 per group).

Daily water consumption and weekly weight was monitored in dams and offspring. TCE exposure did not significantly alter the weights of the dams (data not shown). However, statistically significant body weight differences were detected when mice were weaned at PND21 (Figure 2). Pairwise comparisons revealed that on average, male mice exposed to TCE weighed significantly more than female controls (*p* = 0.008) and TCE-exposed females (*p* = 0.004); 30.6 vs. 22.5 g and 21.84 g, respectively. This difference was not reflected when male groups were compared. At study terminus (PND49) there were no within group differences among males and females regardless of TCE exposure. However, control males weighed significantly more than control females (*p* = 0.009) and TCE-exposed females (*p* = 0.01); 35.7 vs. 29.6 g and 29.8 g, respectively. Overall, male mice weighed more than female mice and this effect did not appear to be influenced by TCE. This result is reflected in Table 1 that shows the *p*-values associated with the two-factor ANOVA analysis that demonstrated a statistically significant main effect of sex on body weight.

**TABLE 1:** Two-factor ANOVA: Effects of sex and TCE treatment and their interaction.

	Interaction	Sex	TCE
Body weight (PND21)	0.20	**0.0004**	0.39
Body weight (PND49)	0.21	**0.001**	0.25
CD4 T cell cytokines			
IFN-γ	0.06	**0.002**	**0.03**
IL-2	0.11	**0.005**	**0.04**
IL-4	**0.002**	**0.0002**	**0.0009**
TNF-α	0.28	**0.01**	**<0.0001**
Liver mRNA expression			
* Il6r*	0.19	0.94	**0.006**
* Gp130*	0.70	0.34	**0.008**
* Egr1*	0.11	0.46	**0.005**
* Ccl5*	**0.0095**	**<0.0001**	**0.05**
* Ccl2*	**0.91**	**0.05**	**0.004**
* Il1b*	0.06	**<0.0001**	0.24
* Tgfb*	0.22	**0.005**	0.06
* Tnfa*	**0.02**	**0.01**	0.06
* Ki67*	**<0.0001**	**<0.0001**	**<0.0001**
* Cyclind1*	**0.04**	**<0.0001**	**0.02**
* Cyp2e1*	0.06	**0.0002**	**0.03**
Cyp2E1 protein	0.69	**<0.0001**	0.1784
Gut cytokines			
IL-3	0.40	**0.0001**	**0.04**
IL-2	**0.03**	**0.06**	0.56
IL-10	**0.004**	**0.934**	**0.01**
IFN-γ	0.14	**0.002**	**0.01**
Eotaxin	0.06	0.08	**<0.0001**
KC	**0.01**	0.07	0.25
GM-CSF	0.89	0.91	**0.0001**
MIP1α	0.35	**0.02**	0.23
IL-1β	**0.02**	0.30	0.44
IL-5	0.56	0.14	**0.006**
IL-4	0.08	0.87	0.19
IL-1α	0.21	0.09	0.88
IL-6	0.45	0.96	0.10
IL-12p40	0.36	0.78	0.59
IL-12p70	0.44	0.23	0.93
IL-13	0.18	0.07	0.18
IL-17	0.23	0.31	0.32
MCP-1	0.13	0.25	0.94
RANTES	0.98	0.08	0.44
TNF-α	0.81	0.16	0.92
Microbiota			
Universal	0.48	**0.04**	0.79

TCE, trichloroethylene. Bold values indicates that the comparison is statistically significant, p < 0.05.

### Trichloroethylene Treatment Mitigates CD4 Proinflammatory Cytokine Production

**FIGURE 3:**
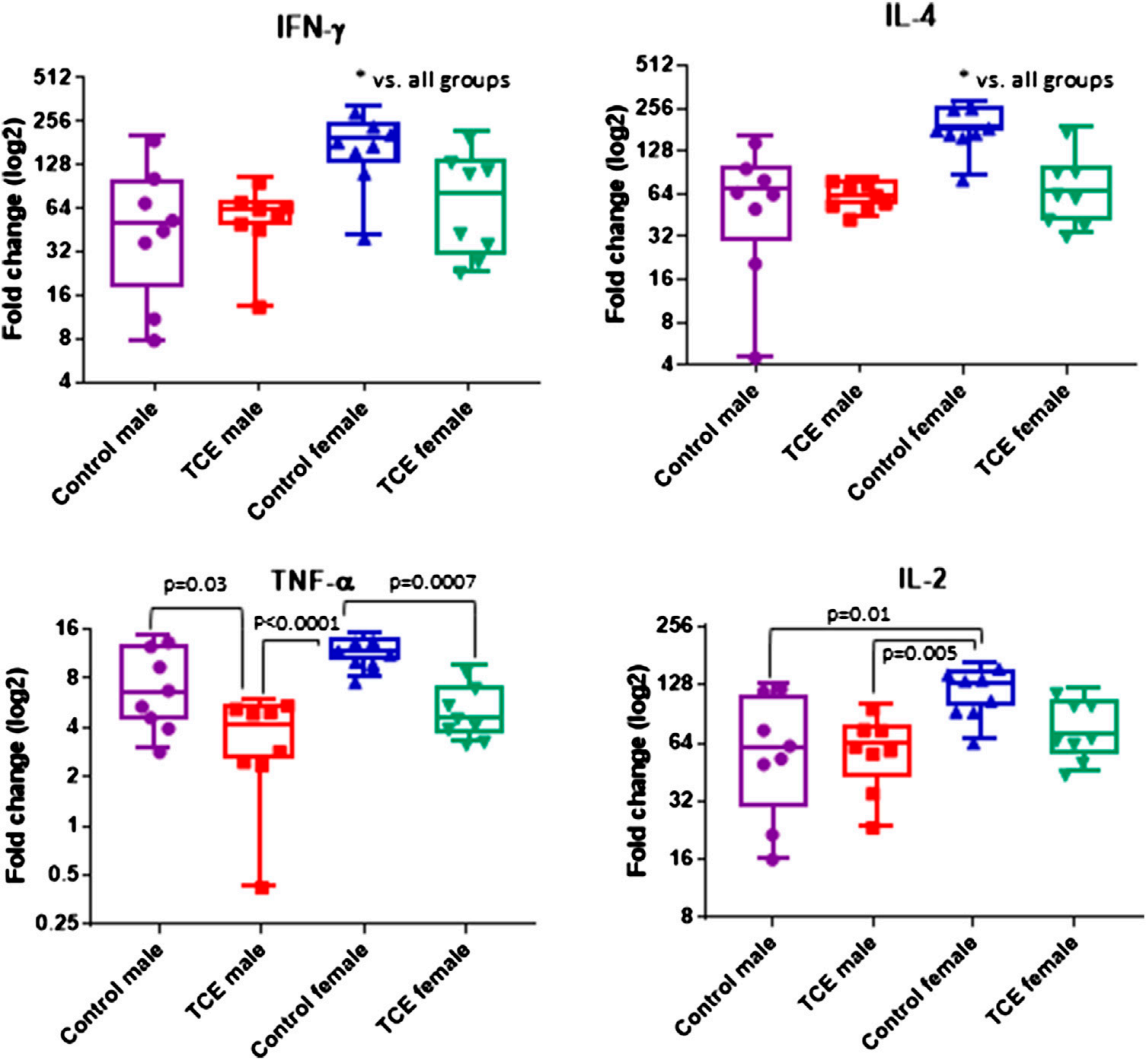
Splenic CD4 cells were isolated and activated as described in the *Materials and Methods* section. Cells were harvested and processed for RNA and cytokine expression was measured by qRT-PCR. Numbers in the bar graphs represent mean (SD) fold-change values relative to unstimulated CD4 cells and normalized by EEF2 housekeeping gene. Data were analyzed by two-way ANOVA to test for interaction and main effects of the two variables [trichloroethylene (TCE) and sex] and reported in **Table 1**. Shown in the graphs are *p*-values for the pairwise Tukey’s *post hoc* analyses comparing eight mice per each group.

CD4^+^ T cells isolated from the four groups of mice were activated *in vitro* and examined for mRNA encoding for CD4 cytokines, IFN-γ, IL-4, TNF-α, and IL-2. CD4 cells from female mice in the control group had increased levels of both *ifng* and *il4* compared to all other groups (Figure 3). However, TCE decreased this effect. When samples from each of the four groups were compared, *ifng* elicited from CD4 cells from TCE-exposed female and male mice, as well as male controls was decreased 2.0-, 3.0-, and 2.7-fold, respectively, compared to female controls. Interestingly, a similar pattern was observed for the ^2^Th cytokine, IL-4 with a mean decrease of 2.4-, 2.9-, and 2.7-fold, respectively, compared to female controls. In contrast, within group differences were observed for *tnfa* mRNA. TCE exposure decreased *tnfa* expression by 2.1- and 2.2-fold in females and males relative to their respective control counterparts. For *il2* expression, both control and TCE-exposed male groups were only statistically significantly lower by 1.79- and 1.92-fold, respectively, when compared to female controls. The results of the two-factor ANOVA in Table 1 showed a statistically significant interaction effect for IL-4, and there were statistically significant main effects of sex and TCE exposure for all cytokines. TCE appeared to mitigate cytokine production at varying degrees for each cytokine, and at least for *ifng*, *il4*, and *il2* control females tended to produce higher levels of CD4 cytokines compared to control males.

### Sex- and Trichloroethylene-specific Alterations in mRNA Expression in Liver

Earlier occurring hepatic events related to inflammation and repair that often precede AIH-like pathology have been found in younger female MRL^+/+^ mice after a period of developmental exposure (Blossom et al., 2018). As shown in Table 2, females expressed higher levels of the proinflammatory chemokine [(C-C motif ligand 5 (ccl5); also known as RANTES] relative to males regardless of TCE exposure, and TCE statistically significantly increased the expression of *ccl5* by 1.4-fold in female mice relative to female controls. Likewise, the expression of *il1b* was increased with TCE-by 1.38-fold in female mice relative to female controls. Evidence of a TCE-enhancing effect was not apparent in males. As for *tnfa*, levels in the liver of TCE-exposed females were higher compared to control males, regardless of TCE exposure, and TCE enhanced expression of this cytokine by 1.8-fold in TCE females relative to control females. As for *tgfb*, control females were higher than males regardless of TCE exposure. However, any treatment related effect in females was not significantly different.

**TABLE 2:** TCE increased liver cytokine/chemokine expression.

	Control male (1)	TCE male (2)	Control female (3)	TCE female (4)	(1) vs. (2)	(1) vs. (3)	(1) vs. (4)	(2) vs. (3)	(2) vs. (4)	(3) vs. (4)
*ccl2*	0.85 (0.32)	0.71 (0.50)	1.60 (0.53)	2.21 (0.89)	0.12	0.46	**0.005**	0.85	0.55	0.16
*ccl5*	0.94 (0.32)	0.78 (0.31)	1.96 (0.87)	3.04 (0.92)	0.95	**0.01**	**<0.0001**	**0.004**	**<0.0001**	**0.009**
*tnfa*	1.07 (0.63)	0.97 (0.43)	1.13 (0.56)	2.04 (0.62)	0.98	0.99	**0.01**	0.95	**0.006**	**0.01**
*il1b*	0.85 (0.32)	0.71 (0.50)	1.60 (0.54)	2.22 (0.89)	0.95	**0.05**	**0.0002**	**0.02**	**<0.0001**	0.15
*tgfb*	0.90 (0.18)	0.78 (0.37)	1.61 (0.78)	1.08 (0.45	0.96	**0.03**	0.87	**0.008**	0.61	0.13

TCE, trichloroethylene. Bold values indicates that the comparison is statistically significant, p < 0.05.

**FIGURE 4:**
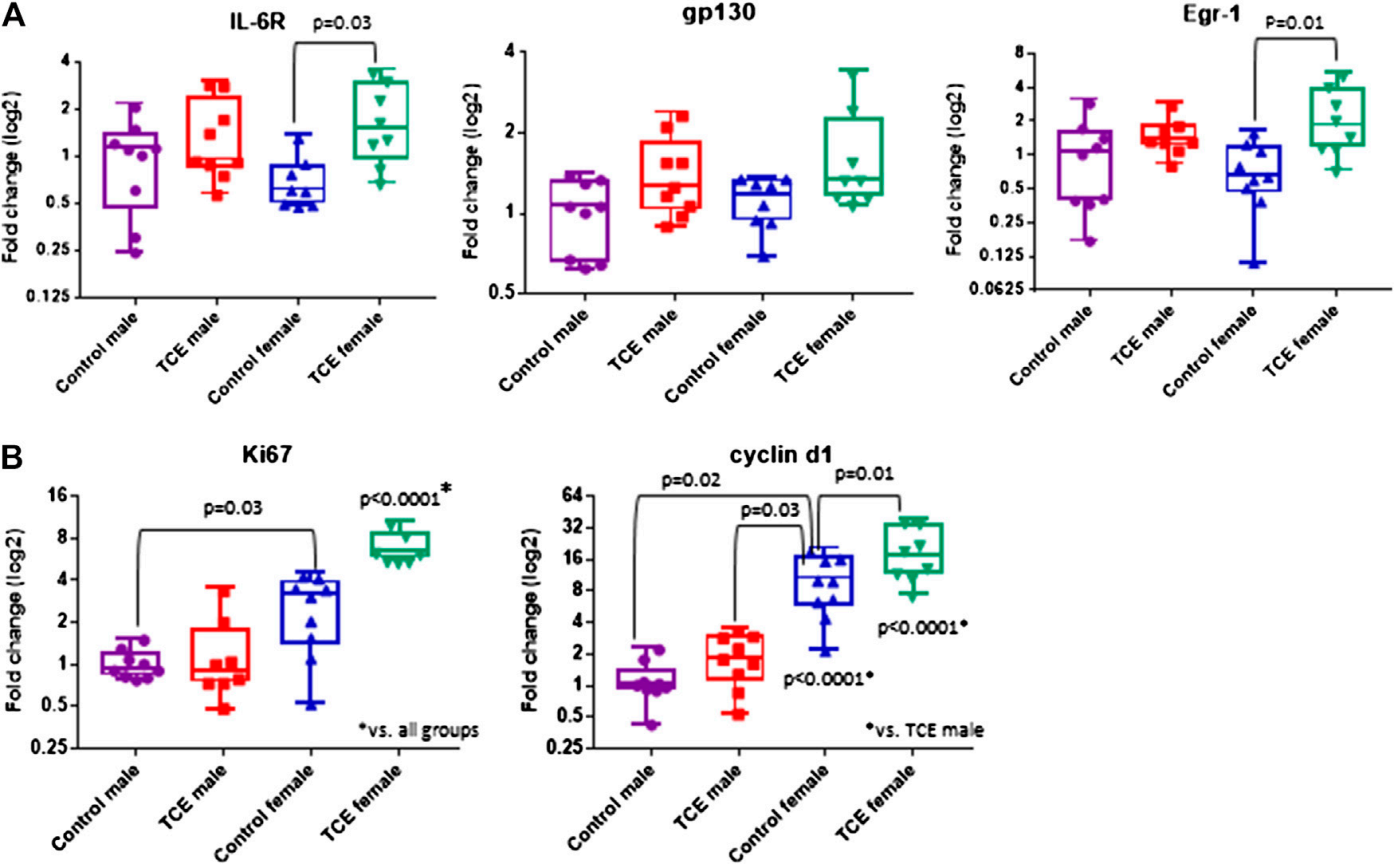
Trichloroethylene (TCE) enhances IL-6 signaling and proliferation markers in the liver of female mice. Expression of liver genes were measured by qRT-PCR as described in the *Materials and Methods* section. Results are represented in the graphs as mean (SD) fold-change. Data points represent individual mice. Data were analyzed by two-way ANOVA to test for significant interaction and main effects between the two variables (TCE and sex) as shown in **Table 1**. Shown in the graphs are *p*-values for the six pairwise Tukey’s *post hoc* comparisons (*n* = 8 mice per group).

Components of the IL-6 signaling complex were also assessed at the mRNA level in liver. As shown in Figure 4A, TCE exposure increased expression of *il6r* in female mice by 2.5-fold relative to their respective controls. Likewise, *egr1* followed a similar pattern in TCE-exposed females with a mean 3.0-fold increase relative to female controls. None of these responses were observed in males. Likewise, *gp130* levels were not statistically significant among the groups. Based on these findings and two-factor ANOVA results (Table 1), female mice exposed to TCE showed the greatest alterations in the expression of liver genes important in inflammation and repair/regeneration.

Because inflammation and liver regeneration and repair are linked to increased proliferation and cell cycle progression, Ki-67 and Cyclin D1, were assessed in the liver at the mRNA level. As shown in Figure 4B, *ki67* transcripts were increased in female controls relative to male controls by 2.6-fold. Liver expression of *ki67* in TCE exposed female mice was similarly enhanced by 2.5-fold relative to female controls. This increase was even more evident when *ki67* expression in TCE-exposed females were compared to relative to control males (6.5-fold increase) and TCE exposed males (5.2-fold increase).

Cyclin D1, an indicator of cell cycle progression that is important in liver signaling and disease was also assessed (Núñez et al., 2017). *Cyclind1* mRNA in liver was significantly increased by 8.7-fold in female controls relative to male controls (Figure 4B). An even greater effect was detected in TCE-exposed females with TCE exposure compared to both control males (13.5-fold) and TCE-exposed males (9.8-fold). Mean *cyclind1* expression was not significantly different between male groups. However, in the female groups, TCE increased expression of *Cyclind1* by 1.91-fold compared to female controls. Thus, similar to inflammation/repair genes, liver levels of proliferation markers were enhanced by a greater degree in females relative to males, and this effect was enhanced by TCE exposure. The two-factor ANOVA analysis revealed significant interaction and main effects among all liver mRNAs tested (Table 1).

**FIGURE 5:**
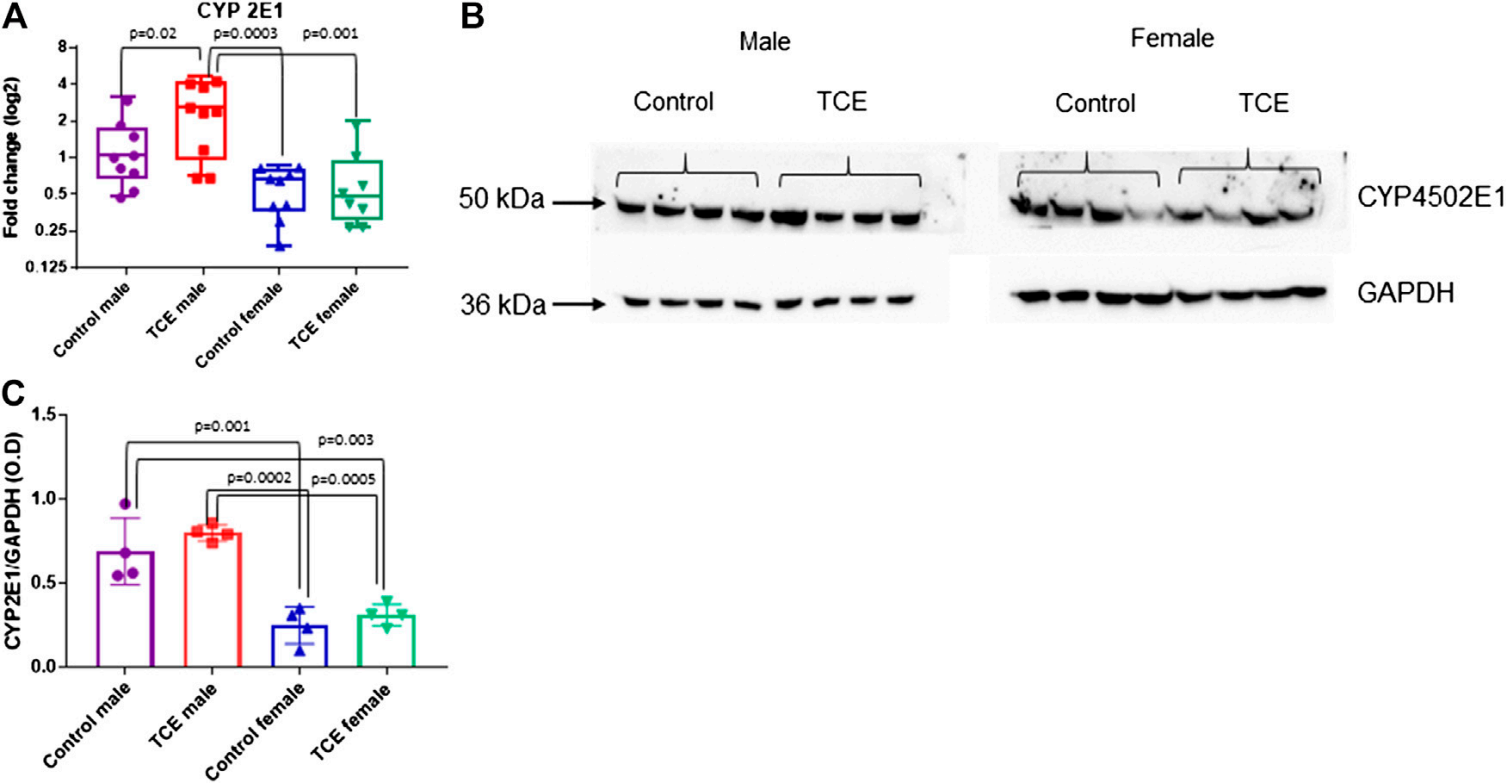
Levels of Cytochrome ^450^P 2E1 were higher in males relative to females. CYP2E1 was measured at the mRNA and protein level by qRT-PCR and western blotting as described in the *Materials and Methods* section. **(A)** Results are represented in the graph as mean (SD) fold-change. Data points represent individual mice (*n* = 8 per group). **(B)** Protein lysates were separated by SDS-PAGE and probed with polyclonal antibodies to CYP2E1 and GAPDH as described in the *Materials and Methods* section in individual mice. **(C)** The values shown in the graph are the mean (SD) OD levels (*n* = 4 per group). Data were analyzed by the two-way ANOVA to test for significant interaction and main effects between trichloroethylene (TCE) and sex as shown in **Table 1**. Shown in the graphs **(A,C)** are *p*-values for the six pairwise Tukey’s *post hoc* comparisons.

Next, we investigated whether the striking differences between males and females in the expression of liver genes were linked to sex differences in the expression of the enzyme that metabolizes TCE. Cytochrome P4 isoform 2E1 (CYP2E1) is the major contributor to the oxidation of TCE in the liver. In a recent pharmacokinetic study, TCE-induced expression in CYP2E1 in liver of female mice to a greater degree compared to males (Luo et al., 2018b). Thus, more efficient enzyme activity by males may help explain the reduced sensitivity toward liver markers compared to females. As shown in Figure 5A, CYP2E1 transcript levels were significantly increased by TCE exposure in males compared to male controls by 2.0-fold. In contrast, there were no treatment-related effects in females. Female mice, regardless of TCE exposure, had significantly lower levels of CYP2E1 compared to TCE-treated males (4.3-fold and 3.4-fold; control female and TCE-exposed females, respectively).

CYP2E1 levels are regulated post-transcriptionally and mRNA may be a less sensitive assay to predict levels of this enzyme based on compensatory mechanisms (Tremmel et al., 2016). Western blots to detect protein levels were conducted in liver lysates and probed with antibody specific for CYP2E1. As shown in Figure 5B, CYP2E1 protein levels detected at around 50–55 kDa appeared to be higher in males relative to females. When CYP2E1 protein levels were normalized to GAPDH and averaged among the groups (Figure 5C), female mice, regardless of TCE treatment, had lower levels of CYP2E1 relative to males, regardless of treatment. However, the TCE effect in males disappeared at the protein level. Likewise, the two-factor ANOVA analysis revealed main effects of sex, but not treatment (Table 1).

### Differential Sex and Trichloroethylene Effects on Mucosal Cytokines and Microbial Composition in the Gut

**FIGURE 6:**
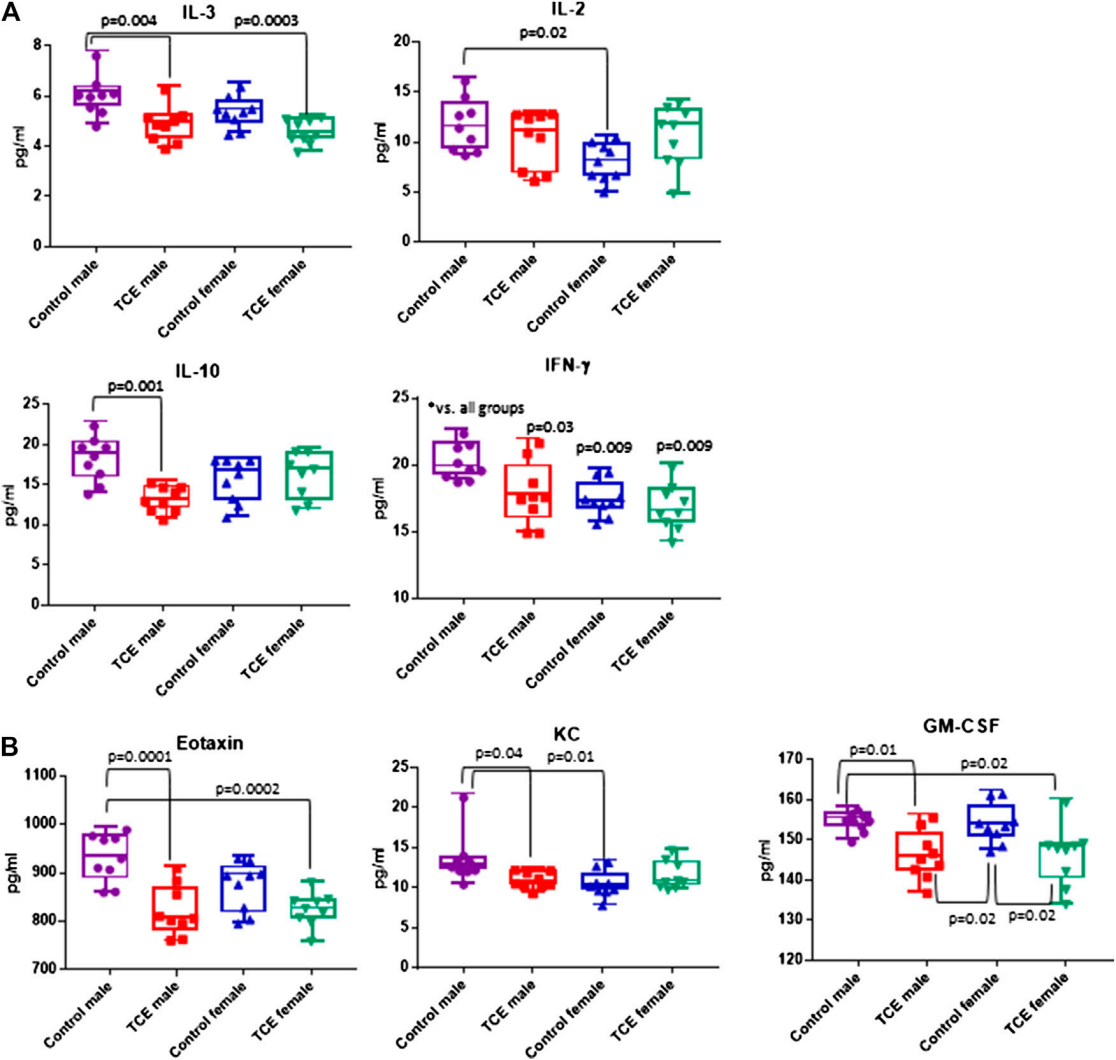
Ileal tissue was harvested from offspring at study terminus. Protein was extracted and multiplex cytokine assay was conducted as described in the *Materials and Methods* section. Means (SD) are represented in the graphs (pg/ml). Data points represent individual mice. Data were analyzed by two-way ANOVA to test for significant and main effects between the two variables [sex and trichloroethylene (TCE)]. Shown in the graphs are *p*-values for the six pairwise Tukey’s *post hoc* comparisons (*n* = 8 per group).

A panel of 20 cytokines and chemokines were assessed in ileal gut tissue, a region that includes Peyer’s patches where the intestinal bacteria that reside there are most closely linked to immune system function (Geuking et al., 2014; Hashiguchi et al., 2015). As shown in Figure 6A, IL-3 was significantly decreased by ∼20% in TCE-exposed males and by ∼24% in TCE-exposed females relative to control males, but not control females. As far as IL-10, there was a mean decrease of ∼28% between TCE-exposed males vs. male controls. IL-2 levels only differed between control groups with a ∼32% decrease in females relative to males. IFN-γ was significantly lower in all groups compared to control males by ∼12%, ∼14%, and ∼17% (TCE male, control female, and TCE female, respectively).

The chemokines, Eotaxin and KC, and growth factor, GM-CSF followed a similar pattern (Figure 6B). Mean levels of Eotaxin from control males were ∼12% higher than TCE-exposed males and ∼11% higher than TCE-exposed females. Likewise, mean levels of KC from control males were ∼20% higher than TCE exposed males, and ∼23% higher than control females. Both male and female TCE groups had significantly lower GM-CSF levels than their control counter parts regardless of sex (∼5–6% decrease). Although TCE appeared to mediate an overall decrease in these mediators, there were a number of cytokines, chemokines, and growth factors that were not significantly different among the groups as shown in Table 1. Thus, unlike what was observed in the liver, the ileal mucosa presented significant TCE-mediated effects in males rather than females.

**FIGURE 7:**
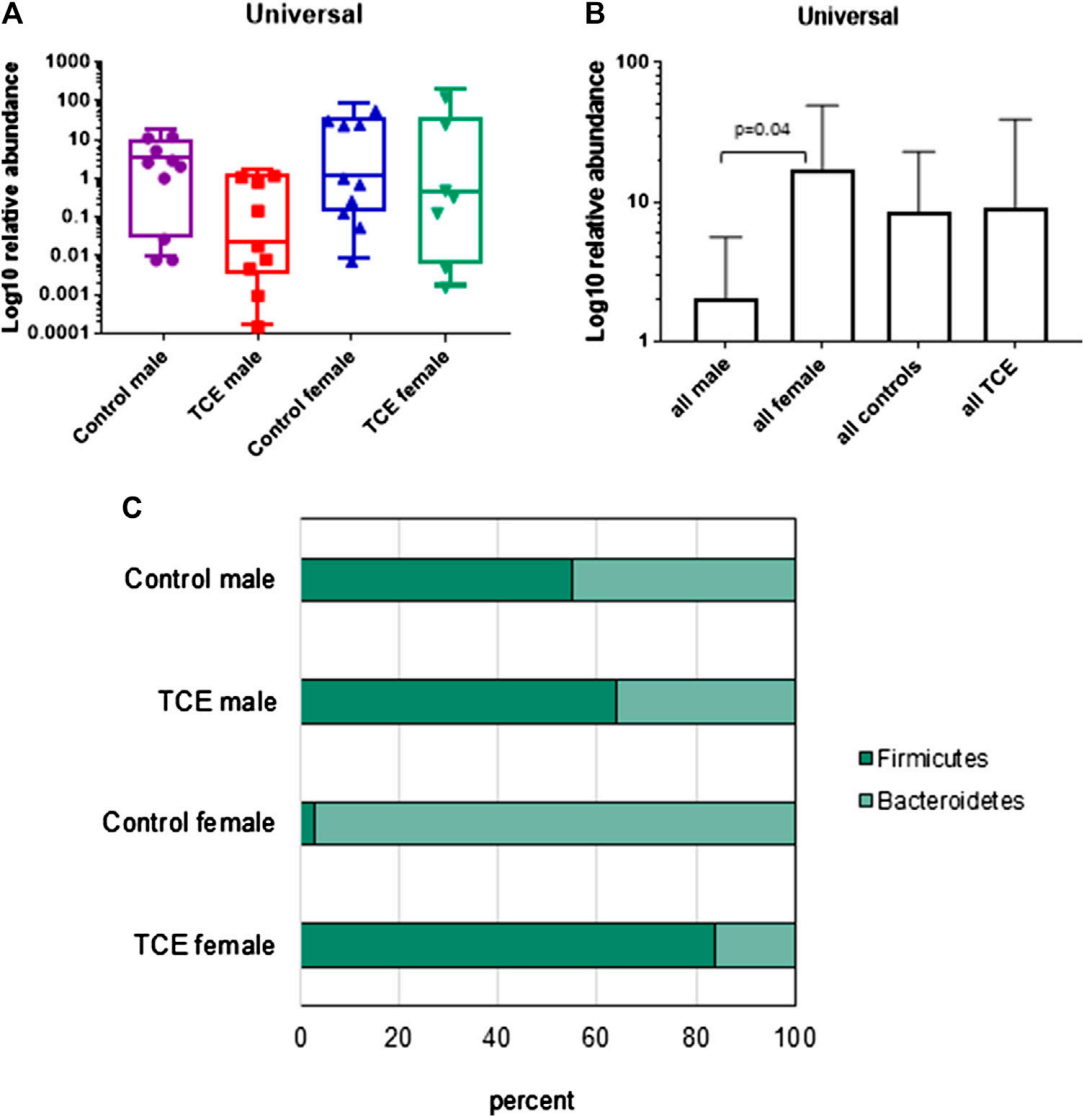
Trichloroethylene (TCE) altered the composition of the gut microbiome. Genomic DNA from ileal tissue was extracted from 10 mice per treatment group, and real-time PCR was used to assess the Ct value of each sample. B-Actin was used as a housekeeping gene. Mean (SD) fold-change (log 10) values for bacterial species of ileal region of the gut are shown. The results show **(A)** the expression of genes represent the entire bacterial population (Universal), **(B)** the data represent the mean (SD) fold change of universal bacteria comparing all males vs. all females and all TCE exposed animals vs. all control animals **(C)**. The ratio of the two phyla (*Bacteroidetes* and *Firmicutes*) are presented in a 100% stack bar diagram, in which each assessed phylum shows the contributions to the sum of the two predominant bacterial phyla.

It is well established that the intestinal mucosal immune system and the cytokines they produce are in close interaction with gut microbiota. Any modification in the balance between microbiota and gut immunological responses may trigger inflammatory disorders (Cianci et al., 2018). Thus, the mucosa-associated bacteria in the ileal region were examined. The results showed a large inter-mouse variation. The abundance of bacterial phyla, including total bacterial population as measured by the expression of universal 16 s RNA were not statistically different when all groups of mice were compared (Figure 7A). Because of the variation, we conducted a different comparison that can be viewed as a type of *post hoc* test, where the combined mean of the two non-treated groups (e.g., male and female) can be compared to the two TCE-exposed groups as described previously (Blossom et al., 2018). This comparison of the relative abundance of bacteria was considerably decreased in all males relative to females regardless of TCE exposure (Figure 7B). We then conducted another analysis where we measured the ratio of Gram-positive phylum (*Firmicutes*) or Gram-negative phylum (*Bacteroidetes*) with regard to TCE and/or sex. Figure 7C represents a 100% stack column for the abundance of bacteria representing these phyla. Interestingly, control female mice were predominantly colonized with *Bacteroidetes* (97%), whereas the total population of control males represented only 45% of this phylum. TCE exposure in females induced a shift in the abundance of *Firmicutes* (∼96% increase). In males, the shift from *Bacteroidetes* to *Firmicutes* also increased with TCE exposure (∼14% increase), but the effect was not as striking as the shift observed in females. Taken together, the alterations observed in gut microbiome appeared to be greatest in female mice and attributable to TCE exposure in both sexes.

## Discussion

A common notion is that autoimmune diseases are predominant among women. However, autoimmune diseases appear to be rising particularly in certain groups including adult males and adolescents (Dinse et al., 2020). It has been postulated that changes in the environment and/or lifestyle are possibly contributors to this increase. The purpose of this study was to determine whether some of the markers known to be altered after developmental exposure in female MRL^+/+^ mice were also altered in male MRL^+/+^ mice. We predicted that responses would be more robust in females compared to males based on their enhanced susceptibility to autoimmunity observable in females in this strain as well as in humans.

We and others have exclusively used female MRL^+/+^ mice for TCE exposure studies *in vivo*. In the absence of TCE, female MRL^+/+^ mice eventually develop nephritis, vasculitis, and T cell-mediated dermatitis with an average mortality of 73 weeks of age. TCE-exposed female MRL^+/+^ mice develop AIH as primary pathology but also inflammatory lesions in lung, pancreas, and kidney (Cooper et al., 2009). It is not known what predisposes this mouse strain toward autoimmunity, but a number of factors are likely at play. CD4^+^ T cells from MRL^+/+^ mice have differential sensitivity to activation signals compared to non-autoimmune strains of mice which may contribute to their autoimmune phenotype (Vratsanos et al., 2001; Lang and Bellgrau, 2002).

The MRL mouse strain has been extensively characterized. Male MRL^+/+^ mice develop a milder form of lupus-like disease compared to female MRL^+/+^ mice, and die on average at 93 weeks of age, which is similar to the lifespan of non-autoimmune-prone mice (Storer, 1966). Since comprehensive studies to evaluate TCE-mediated responses in male MRL^+/+^ mice have not been conducted, an assessment of both sexes was expected to provide insight into the understanding of sex disparity in autoimmunity. We selected certain endpoints that have been consistently shown to be altered by TCE and AIH development in previous investigations including CD4 cytokines (e.g., IFN-γ, markers of liver inflammation, regeneration/repair, and proliferation, as well as intestinal microbiome and cytokines).

In the current study, both male and female MRL^+/+^ mice were chronically exposed to a single concentration of TCE from conception until young adulthood as described (Meadows et al., 2017). It was predicted that females would have more robust effects than males, and that TCE would enhance these responses. For the most part, our predictions were confirmed. Female mice produced higher levels of cytokines from activated CD4^+^ T cells, including IFN-γ, relative to males regardless of TCE exposure. Interestingly, TCE appeared to mitigate this effect. While this result might appear to contradict previous findings, a long-term multi-endpoint evaluation of the IFN-γ response by activated CD4^+^ T cells was evaluated in a previous study where temporal differences in the expression of IFN-γ over the course of TCE exposure was reported (Gilbert et al., 2016). Additional microarray analyses revealed alterations in many immune-related genes. For instance, *ifng* increased after 22 weeks of exposure yet decreased after 40 weeks relative to controls (Gilbert et al., 2017). This biphasic response is consistent with several studies documenting time-dependent inflammatory mediator fluctuations in other autoimmune mouse models (Kuerten et al., 2010). It is also common in humans with autoimmune disease where cytokine fluctuations correlate with an early disease phase followed by a temporary recovery and then clinical relapse (Ryden et al., 2009). Thus, the decreased *ifng* expression by effector/memory CD4^+^ T cells in the current study may be due to a temporary compensatory response which could eventually be overcome after continued exposure. This waxing and waning of responses by CD4^+^ T cells represent one of the limitations associated with studying cytokine response patterns obtained at a single time point.

IFN-γ has been proposed as a regulator of cytokine and chemokine genes in the liver, and is similarly dichotomous in their regulation in a mouse model of fulminant AIH (Milks et al., 2009). A suite of these and other pro-inflammatory markers were examined at the mRNA level. Levels of *tgfb*, *il1b*, *tnfa*, and *ccl5* were increased in females compared to males regardless of TCE exposure, and in most cases, TCE further enhanced these chemokines in TCE-exposed females relative to controls. These results appear to reflect a proinflammatory environment in female livers. Chemokines are increased in many different liver injury models as well as in AIH (Chi et al., 2018), and are important in recruitment of immune cells, including T cells, to the liver. Elevation of CCL5, also known as RANTES, has been linked to human AIH (Czaja, 2014). Thus, the increase of these markers in females rather than in males may help explain the increased female propensity of AIH in humans. Our findings might also mean that males lag behind females in the development of immune pathology. Additional studies where exposure is extended would need to be conducted in order to gain a better understanding of disease progression in males vs. females.

Similar to chemokines, TCE significantly increased mRNA for *egr1* in female, but not male mice. In a contradictory report, TCE exposure reduced EGR-1 in a mouse model of autoimmune-mediated cholangitis (Ray et al., 2017). However, in the MRL model, this complex has previously been shown to be enhanced by TCE early in the course of adult only chronic exposure in female MRL^+/+^ mice (Gilbert et al., 2014). In a recent study, TCE also enhanced mRNA expression of both chemokines and IL-6 complex components in livers of adult female MRL^+/+^ mice following a period of developmental exposure (Blossom et al., 2018). Thus, the differential expression of *egr-1* by TCE among the studies may be due to strain related differences. In addition, the hepatic response to injury may be time-dependent since it is exquisitely regulated involving up- and down-regulation of inflammatory cytokines and growth factors.

The transcription factor EGR-1 regulates the expression of genes required for execution of the wound healing response. Multiple cycles of injury, coupled to incomplete wound healing, can lead to fibrosis. EGR-1 has been reported to be a negative regulator of hepatic fibrosis mediated by the environmental pollutant and liver toxicant, carbon tetrachloride (Pritchard and Nagy, 2010). Interestingly, ethanol-elicited regulation of EGR-1 expression depends on the generation of acetaldehyde, and the absence of EGR-1 diminishes alcohol-induced liver injury (Thomes and Donohue, 2017). Both ethanol and TCE are metabolized similarly (Cichocki et al., 2016). Thus, it appears that EGR-1 acts in a pleiotropic manner and may also promote or suppress liver injury depending on the pathway.

IL-6 is much like EGR-1 in that it can either promote or suppress inflammation. IL-6, important in T cell-mediated liver injury, binds to a complex consisting of gp130, a transmembrane protein, and IL-6 receptor (IL-6R) on the surface of hepatocytes. While an alteration in *gp130* was not observed, TCE enhanced IL-6R mRNA in females, but not males, exposed to TCE.

Response to liver insults are also linked to proliferation and cell cycle progression. TCE-exposed females expressed high levels of proliferation and cell cycle markers, *ki67* and *cyclnd1*, relative to control females and males, regardless of TCE exposure. Like IL-6 related markers and chemokines/cytokines, KI67 and Cyclin D1 dysregulation is an indicator of many different liver conditions including hepatocellular carcinoma (Salama et al., 2019), nonalcoholic fatty liver disease (Dey et al., 2019), chronic alcohol exposure (Chavez et al., 2011), and liver injury (Inomata et al., 2018). The role of these proteins are not well described in AIH, however; experimentally-induced AIH had altered levels of these proliferation markers (Huang et al., 2017). Together, these results indicate that the liver in females, relative to males, are more responsive to liver injury, and TCE enhances these effects.

CYP2E1 is the major liver enzyme responsible for metabolizing TCE. Using a specific inhibitor, it was determined that CYP2E1 ameliorated both CD4^+^ T cell effects and liver pathology (Griffin et al., 2000). These results have been confirmed in female CYP2E1 MRL knockout mice (Wang et al., 2019a). Thus, it was plausible to hypothesize that immune and liver differences observed in females +/− TCE may be due to differences in levels this enzyme in males vs. females. In the current study, male mice had higher levels of CYP2E1 transcripts and protein levels relative to females. However, TCE only further increased these effects at the mRNA level in males. Since CYP2E1 is modified post-transcriptionally, it is unlikely that sex differences in the levels of this enzyme were attributable to the phenotype mediated by TCE. The striking differences in liver markers may be explained by other TCE- and sex-related factors including hormones, and reported differences in responses to oxidative stress and inflammation. Together, these findings have far reaching implications not only for sex disparity in TCE-mediated liver toxicity, but also for immune dysfunction and autoimmune disease.

AIH-like T cell-mediated pathology, including lymphoplasmacytic portal infiltrate and lobular inflammation was not evident (data not shown). Sera was examined for the presence of anti-liver antibodies against microsomal liver protein as a more specific marker of AIH (Gilbert et al., 2017; Blossom et al., 2018). We were unable to detect sex- or TCE-dependent differences in serological markers of anti-liver antibodies or anti-nuclear antibodies (ANAs) among the groups in the current study (data not shown). Thus, this relatively brief duration of exposure in young mice was not associated with autoimmune pathology. This result was not surprising since immune pathology associated TCE exposure is typically detected in older mice after chronic adult or developmental exposure (Griffin et al., 2000; Gilbert et al., 2017). The mice in the current investigation, were only 7 weeks of age at study terminus. A future study will encompass later time points in order to determine whether or not TCE exposure imparts serological or histopathological signs of autoimmunity in males.

Several gut cytokines in female MRL^+/+^ mice developmentally exposed to TCE were altered. This included decreases in IL-3, Eotaxin, GM-CSF, and IFN-γ (Blossom et al., 2018; Khare et al., 2019), and an increase in KC (Khare et al., 2019). Unexpectedly, these differences were more apparent in males rather than females, and treatment related effects were only observed in males except for GM-CSF, where treatment related decreases were observed in both sexes. A decrease in gut cytokines is consistent with what would be expected with a decrease in microbial abundance, which may be due in part to the propensity of microbes to produce short-chain fatty acids which can markedly reduce innate immune activation in the gut (Trapecar et al., 2020). It is not clear why females had fewer *Firmicutes* than males to begin with, yet showed a greater increase in this population with TCE exposure. Perhaps these inflammatory cells and their mediators along with microbial products are increasingly translocated to the liver due to greater liver injury markers in females relative to males. Another plausible explanation for the disparity in the male and female microbial population could be the intrinsic presence of sex-specific microbiota composition (Org et al., 2016). There are many similarities between our model and alcoholic liver disease which are both linked to decreased abundance of B*acteroidetes* relative to *Firmicutes* (Baker and Baker, 2020). In autoimmune models, the microbiome is altered with increased lactobacilli (a *Firmicute*) increasing throughout the course of the disease in NZB/NZW ^1^F lupus-prone mice (Mu et al., 2017; Luo et al., 2018a).

A limitation was the study used a single concentration of TCE at one time point and the mice were examined at a relatively young age. Mice administered water containing 0.5 mg TCE/ml were exposed to levels of 145 mg/kg/day (via maternal exposure through gestation and lactation) and 61 and 68 mg/kg/day (male and female offspring, respectively) through direct exposure of the drinking water for approximately 4 weeks; from weaning until the mice were euthanized at PND49 or ∼7 weeks of age. The dose of TCE from direct exposure (PND21-PND49) was slightly lower than the current 8 h PEL of 100 ppm or ∼76 mg/kg/day. The US EPA Maximum Contaminant Level (MCL) for drinking water is 5 μg TCE/L, although exceedance of the MCL has been reported (ATSDR, 2014). The TCE dose used in the present study is several orders of magnitude higher than would be expected with environmental exposures from oral ingestion.

Our findings have important implications for human health, and understanding sex disparity in responses to TCE. Overall, females produced more robust responses in terms of immune- and liver-toxicity, and changes to the microbiome. Future research will explore a range of doses and later in life time points to examine tissue pathology, and mechanisms behind the sex differences revealed in the current study.

## Data Availability Statement

The raw data supporting the conclusions of this manuscript will be made available by the authors, without undue reservation, to any qualified researcher.

## Ethics Statement

The animal study was reviewed and approved by University of Arkansas for Medical Sciences Institutional Animal Care and Use Committee.

## Author Contributions

SK assisted with manuscript writing, data analysis, and experimental design; KG and MA contributed by conducting gut cytokine and microbiome assays as well as data analysis; and SB was responsible for overall experimental design, manuscript writing and preparation, and conducted data analysis.

## Disclaimer

The findings and conclusions presented in this manuscript are those of the authors and do not necessarily represent the views of the US Food and Drug Administration.

## Funding

This work was supported by a grant from the National Institutes of Health (NIEHS-R01ES021484).

## Conflict of Interest

The authors declare that the research was conducted in the absence of any commercial or financial relationships that could be construed as a potential conflict of interest.
