# Supplementary material for: Sex-Dependent Effects on Liver Inflammation and Gut Microbial Dysbiosis After Continuous Developmental Exposure to Trichloroethylene in Autoimmune-Prone Mice
Source: Front Pharmacol. 2020 Oct 29;11:569008. doi: 10.3389/fphar.2020.569008 (PMC7673404; doi:10.3389/fphar.2020.569008)
Supplement: Supplementary file 1 [file Table1_v2.docx]

Supplementary Table 1. Primers used for the real-time gene expression of bacteria

| **Gene** | **Sequence** | **Amplicon (bp)** |
| --- | --- | --- |
| *Firmicutes* | 5'-GGAGYATGTGGTTTAATTCGAAGCA -3' | 126 |
|  | 5'-AGCTGACGACAACCATGCAC-3' |  |
|  |  |  |
| *Bacteroidetes* | 5'-GGARCATGTGGTTTAATTCGATGAT-3' | 126 |
|  | 5'-AGCTGACGACAACCATGCAG-3' |  |
|  |  |  |
| Universal primer | 5'-ACTCCTACGGGAGGCAGCAG-3' | 200 |
|  | 5'-ATTACCGCGGCTGCTGG-3' |  |
